# DNA polymerase theta (Polθ): a novel candidate for targeted cancer therapy

**DOI:** 10.1080/15384047.2026.2703892

**Published:** 2026-07-28

**Authors:** Bo Zhou, Zhixin Wang, Jun Qi, Fengke Liang, Haohui Liu, Yunqi Li

**Affiliations:** a Department of Gastroenterology, The 944th Hospital of PLA Joint Logistics Support Force, Jiuquan, China; b Tianjin International Travel Healthcare Center, Tianjin, China; c Department of Gastroenterology, First Medical Center, Chinese PLA General Hospital, Beijing, China

**Keywords:** Polθ, MMEJ, DNA damage repair, synthetic lethality, targeted therapy

## Abstract

DNA double-strand breaks (DSBs) are the most severe DNA damage, and defective repair can lead to apoptosis or malignant transformation. DSBs are mainly repaired by nonhomologous end joining (NHEJ) and homologous recombination (HR), while microhomology-mediated end joining (MMEJ) serves as a backup pathway. Since DNA polymerase theta (Polθ) is essential for MMEJ, this pathway is also named Polθ-mediated end joining. Polθ is barely expressed in normal tissues but overexpressed in many cancers, making it a promising therapeutic target. In recent years, Polθ inhibitors and related therapeutic strategies have emerged rapidly, with clinical trials underway. This review summarizes the structure, function and expression of Polθ in tumorigenesis, highlights synthetic lethal strategies, drug development and clinical translation, and discusses current limitations and future directions for cancer research.

## Article highlights

- Polθ-mediated microhomology-mediated end joining (MMEJ) promotes genomic instability and facilitates mutation accumulation, thereby leading to cancer initiation and progression.
- Polθ expression is significantly upregulated in various cancer types and markedly correlates with cancer prognosis.
- DNA damage repair pathways and some proteins can regulate Polθ expression and mediated microhomology-mediated end joining (MMEJ) activity in cancer.
- Polθ inhibitor have appeared sequentially and clinical trials targeting Polθ are being gradually launched.
- Polθ is a highly attractive therapeutic target and synthetic lethal strategies targeting Polθ in cancer have been continuously explored.

## 1. Background

Cancer is a major global cause of mortality. In 2022, there were around 20 million new cancer cases and 9.7 million cancer-related deaths globally.^1^ By 2050, the annual number of new cancer cases worldwide is projected to reach 35 million.^1^ Radiotherapy and chemotherapy are widely used as primary cancer treatments, markedly improving patient survival rates and making substantial contributions to the fight against cancer. However, tumor heterogeneity has exacerbated challenges such as drug resistance and radioresistance induced by long-term radiotherapy and chemotherapy. Some patients do not experience notable improvement in survival prognosis, and they bear substantial economic burdens.^2^ ^,^ ^3^ Radiotherapy and chemotherapy not only target tumor cells but also harm normal cells, causing side effects like alopecia and anemia.^2^ Extended treatment duration can elevate the risk of diabetes and cardiovascular diseases.^4-11^ To address cancer resistance to radiotherapy and chemotherapy, improve therapeutic efficacy, and minimize side effects, it is essential to identify more selective and effective tumor treatment options.^12^

**Figure 1.**
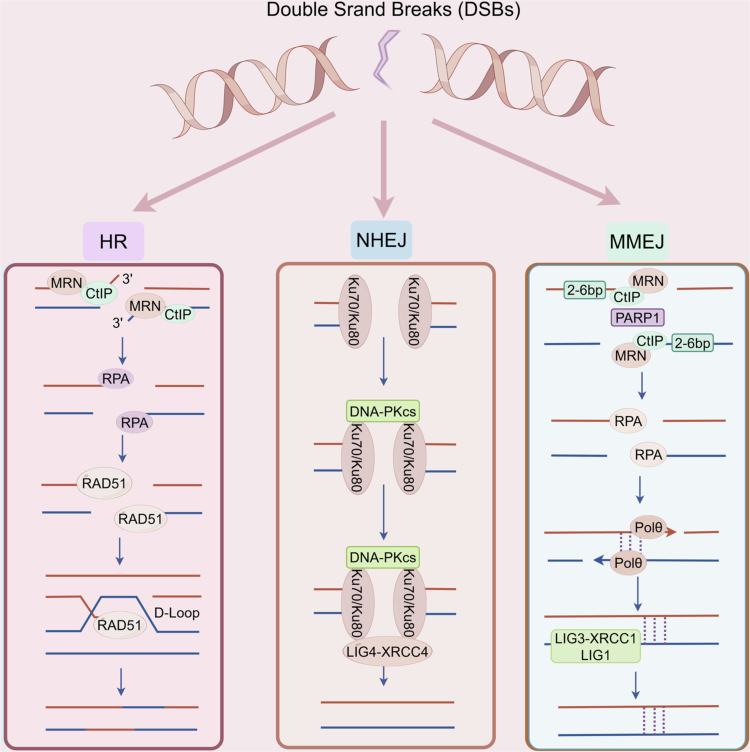
Three main repair pathways of DSBs. DSBs can be repaired by HR, NHEJ, and MMEJ. The three DSB repair pathways exhibit distinct cell cycle dependencies: NHEJ predominates in the G1 phase of the cell cycle, HR is restricted to S/G2 phases, and MMEJ is the predominant DSB repair pathway during the M phase. HR is initiated by the MRN complex and CtIP to produce 3′ ssDNA, which is coated by RPA. RAD51 then displaces RPA to form D-loop formation and DNA synthesis. In the NHEJ pathway, Ku70/80 binds to DNA ends and recruits DNA-PKcs. DNA-PKcs then recruits LIG4-XRCC4 to ligate the DNA ends. PARP1 binds to the DSB site and facilitates MMEJ repair. MMEJ and HR share the initial DNA end resection step to generate 3′ ssDNA, which is coated by RPA. Polθ displaces the RPA and anneals exposed microhomology sequences. Then Polθ initiates DNA synthesis at the microhomology site, and subsequently, the newly synthesized DNA is ligated by LIG3-XRCC1 or LIG1. Abbreviations: DSBs, double-strand breaks; HR, homologous recombination; NHEJ, nonhomologous end-joining; MMEJ, microhomology-mediated end joining; MRN, MRE11-RAD50-NBS1; CtIP, CtBP-interacting protein; ssDNA, single-strand DNA; RPA, replication protein A; Polθ, DNA polymerase theta; DNA-PKcs, DNA-dependent protein kinase catalytic subunit; LIG4, DNA ligase IV; XRCC4, protein X-ray repair cross-complementing 4; PARP1, poly (ADP-ribose) polymerase 1; LIG3, DNA ligase III; XRCC1, protein X-ray repair cross-complementing 1; LIG1, DNA ligase I.

Cells continuously accumulate DNA damage due to various physical and chemical factors. To maintain genome stability, cells possess an advanced DNA damage repair (DDR) system to address diverse types of lesions.^13^ ^,^ ^14^ Double-strand breaks (DSBs) are the most lethal form of damage. Their appropriate repair prevents excessive damage accumulation, thereby preventing cell death or tumorigenesis.^15^ ^,^ ^16^ The repair of DSBs is vital for cellular growth, development, and regulation of cell fate. Cells primarily employ repair mechanisms such as nonhomologous end joining (NHEJ), homologous recombination (HR), and microhomology-mediated end joining (MMEJ) (Figure 1).^17^ Impaired DSB repair functions result in unrepaired damage, leading to increased genome instability, which facilitates the initiation and progression of cancer.^18^ ^,^ ^19^ DSBs have a dual role: they may lead to carcinogenesis but can also be utilized in cancer treatments.^15^ ^,^ ^17^ ^,^ ^20^ The principle underlying radiotherapy and chemotherapy in cancer treatment is to induce excessive DSBs, thereby initiating the death of cancer cells.

Unlike the precise repair mechanisms of HR and NHEJ, MMEJ is marked by high error-proneness. MMEJ employs microhomologous sequences flanking the damage site for repair, leading to loss of partial genetic information.^21^ Moreover, DNA polymerase theta (Polθ), the principal protein of the MMEJ pathway, is susceptible to introducing mutations, insertions, and deletions during DNA synthesis.^21-23^ Polθ is often minimally expressed or absent in normal cells due to its tendency to alter genetic information. Consequently, MMEJ serves as an inactive backup pathway for DSB repair.^21^ ^,^ ^23^

Notably, the accumulation of gene mutations induced by MMEJ during DDR can drive cellular carcinogenesis, resulting in aberrantly elevated expression levels of Polθ and its associated MMEJ repair capabilities during cancer initiation and progression. Polθ's marked differential expression between normal and cancer cells highlights its potential as a promising target for cancer therapy.^24^ The prospect of treating tumors with specific Polθ inhibitors without harming normal cells represents an exciting future direction.

In the past few years, some reviews centering on the structure, enzymatic function, and biological roles of Polθ have been successfully published.^25-27^ Early classic reviews mainly focused on the structural characteristics of the polymerase and helicase domains of Polθ, as well as its core function in MMEJ repair. Several subsequent reviews further extended to the oncogenic effects of Polθ in tumorigenesis. Nevertheless, most reviews only collated key discoveries released prior to 2024.^25-27^ In our review, we systematically integrate the groundbreaking advances regarding Polθ reported between 2024 and 2026, thereby updating the latest research landscape of this field. In addition, this work provides a new comprehensive summary of the regulation of Polθ and the interactions between Polθ and the mismatch repair (MMR) pathway. Collectively, this review fills critical gaps in the temporal coverage and pathway-interaction dimension of previous Polθ reviews, and delivers updated insights to guide follow-up mechanistic and translational investigations of Polθ.

This review systematically presents the structure and function of Polθ, along with the key mechanisms of the MMEJ repair pathway. We further elucidated the correlation between Polθ and cancer, describing the regulatory mechanisms governing Polθ expression in malignancies. Additionally, we outlined synthetic lethal strategies related to Polθ and current advances in the development of Polθ inhibitors. We highlighted Polθ's potential as a cancer therapeutic target and explored possible treatment strategies.

## 2. Polθ and MMEJ

### 2.1. Structure and function of Polθ

The POLQ gene encodes the 290 kDa protein Polθ, which plays a crucial role in MMEJ. Polθ is a multifunctional repair protein with a conserved *N*-terminal helicase domain and a C-terminal polymerase domain.^28^ ^,^ ^29^ Polθ is uniquely characterized among eukaryotic DNA polymerases by its helicase activity.^28^ The helicase domain of Polθ exhibits DNA-dependent ATPase activity that facilitates the removal of RAD51 from single-stranded DNA (ssDNA), thus suppressing HR repair. Additionally, it can remove replication protein A (RPA) from ssDNA ends.^30^ ^,^ ^31^ The polymerase domain of Polθ aids in annealing microhomologous sequences and extending DNA strands, but it is error-prone in DNA synthesis and lacks proofreading ability.^32^ The central domain of Polθ shows low sequence conservation and might regulate activity, as a protein lacking this domain remains functional in MMEJ.^33^

Polθ is a key protein in the MMEJ repair pathway, and its mediated end-joining repair represents one of the pathways for DSB repair.^34^ A differentiating feature of MMEJ from other DSB repair mechanisms is its ability to repair DSB during the mitotic phase.^35-39^ MMEJ requires 3’ ssDNA overhangs at the resected DNA ends, microhomologous regions of 2–6 nucleotides in length, and the presence of Polθ.^40^ The absence of proofreading in Polθ and the concomitant loss of genetic information associated with MMEJ, this repair mechanism is highly prone to inducing mutations.^41^ ^,^ ^42^ Consequently, the MMEJ pathway frequently serves as a supplementary repair pathway, becoming active when NHEJ or HR repair is compromised. In tumor cells exhibiting impaired HR or NHEJ, Polθ expression is markedly upregulated, resulting in enhanced MMEJ activity, as these cells depend on MMEJ for survival. When HR and NHEJ are dysfunctional, Polθ-mediated MMEJ becomes particularly essential.^20^ ^,^ ^43^ However, a previous study has demonstrated that Polθ could be overexpressed in cells with normal DSB repair capacity, implying that defects in other DNA repair pathways may lead to dependence on MMEJ.^22^

Polθ exhibits extremely low or undetectable expression in most normal somatic tissues, which lays a theoretical foundation for the tumor-selective toxicity of Polθ-targeted inhibitors. However, Polθ is broadly conserved across most metazoan and plant species, suggesting that Polθ carries limited yet physiologically irreplaceable functions.^44^ First, Polθ exerts limited yet irreplaceable genome-protective functions across germ cells; loss of Polθ function leads to defective gametogenesis and impaired fertility.^45^ Second, Polθ is required to sustain the genome stability of hematopoietic stem cells under replicative stress, and its deficiency disrupts long-term hematopoietic homeostasis.^46^ Third, Polθ is critical for DNA repair in embryonic stem cells. Zebrafish embryos require Polθ to develop normally upon Cas9-triggered DSBs.^47^ The results in Drosophila demonstrate that Polθ is also crucial for repairing DSBs that occur during development.^48^ Fourth, HR and NHEJ are repressed in mitosis; MMEJ is the sole DSB repair pathway.^35^ ^,^ ^36^ MMEJ activity in M phase is driven by the accumulation of RHINO to promote Polθ recruitment to damage sites for DNA repair. These indispensable physiological roles are critical safety considerations for the clinical application of Polθ-targeted inhibitors, as long-term pharmacological suppression may trigger reproductive toxicity, hematopoietic suppression, or developmental toxicity.

### 2.2. Polθ-mediated MMEJ

Polθ-mediated MMEJ repair primarily involves the following steps (Figure 1). Poly (ADP-ribose) polymerase 1 (PARP1) is crucial for mediating MMEJ repair.^49^ Upon detecting DNA DSB damage, PARP1 binds to the DSB site. Similar to HR, when a DSB occurs, MMEJ repair is activated by the MRE11-RAD50-NBS1 (MRN) complex, which identifies the DSB ends and recruits CtBP-interacting protein (CtIP) to the damage site to initiate end resection.^38^ Post-DNA end resection, 3' ssDNA overhangs are generated on either side of the break, which are subsequently coated by RPA. Polθ is subsequently recruited to the 3' ssDNA overhangs. The helicase domain of Polθ removes RPA proteins from the 3' ssDNA overhangs, aiding the next phases of MMEJ repair.^50-52^ Polθ facilitates the identification of microhomology sequences, including those with a minimum of two base pairs in length. Subsequently, nonhomologous DNA sequences at the 3' ssDNA overhangs are eliminated through nuclease activity.^53^ Polθ anneals the microhomology sequences, which must comprise at least 2 nucleotides in length. Polθ's polymerase domain begins error-prone DNA synthesis at the microhomology site. The newly synthesized DNA is joined by DNA ligase III (LIG3), protein X-ray repair cross-complementing 1 (XRCC1), and DNA ligase I (LIG1).^29^

## 3. The expression and clinical relevance of Polθ in cancer

**Figure 2.**
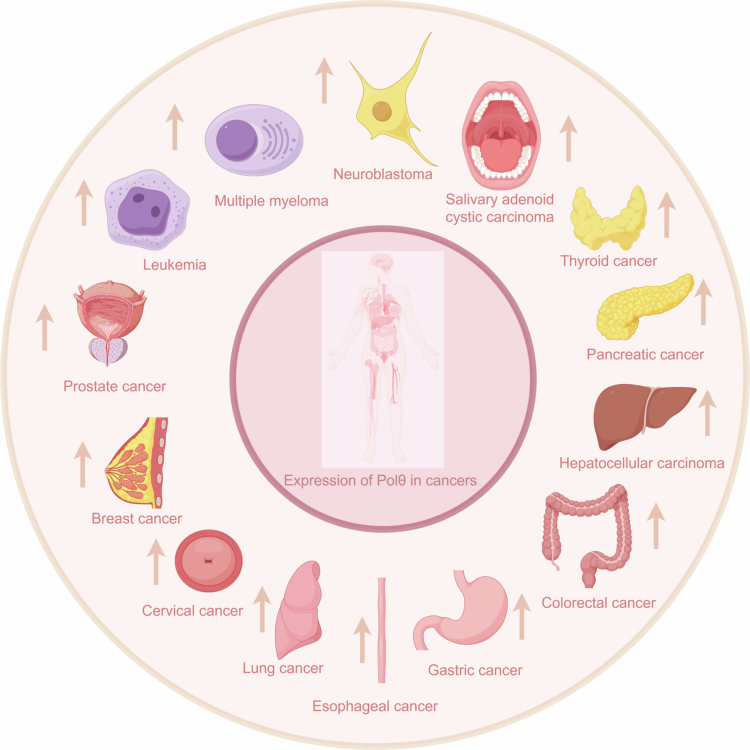
Polθ expression in various cancer types. Polθ expression is significantly upregulated in various cancer types, such as lung cancer, breast cancer, and colorectal cancer, etc. Polθ, DNA polymerase theta.

Genomic instability is a key characteristic of cancer.^18^ ^,^ ^19^ Polθ and Polθ-mediated MMEJ are susceptible to inducing genomic instability, thereby implicating Polθ as closely associated with cancer initiation and progression. Numerous studies reported that Polθ was rarely or not expressed in normal human tissues, whereas its expression was significantly upregulated in various cancer types (Figure 2). In some cancers, Polθ expression was markedly correlated with tumor prognosis (Table 1). These findings collectively elucidate the close relationship between Polθ and cancer.

**Table 1.** Clinical significance of Polθ expression in various cancers.

Cancer type	Polθ expression in cancer	Clinical associations	Reference (PMID)
Breast cancer	High	Poor prognosis	PMID 20624954
Lung cancer	High	Poor prognosis	PMID 14735362
	High	Advanced pathological stage	PMID 31137743
	High	High tumor stage, poor prognosis, increased tumor mutational burden	PMID 36642785
Esophageal cancer	High	Poor prognosis	PMID 34206946
Gastric cancer	High	Poor prognosis	PMID 14735362
	High	Poor prognosis	PMID 41645172
Colorectal cancer	High	Poor prognosis	PMID 14735362
	High	Poor prognosis	PMID 39520627
hepatocellular	High	Poor prognosis	PMID 34517891
carcinoma	High	Poor prognosis	PMID 40215812
	High	High T stage and pathological stage, poor prognosis	PMID 40735692
Pancreatic cancer	High	Poor prognosis	PMID 39435280
Thyroid cancer	High	Dedifferentiation, poor prognosis	PMID 38897886
Salivary adenoid cystic carcinoma	High	Poor prognosis	PMID 36726713
Neuroblastoma	High	Poor prognosis	PMID 40482467
Multiple myeloma	High	Advanced tumor stage, poor prognosis	PMID 41074018
Prostate cancer	High	Poor prognosis	PMID 32877750

In breast cancer, Fanny Lemee et al. analyzed the gene expression profiles of patients with breast cancer from two independent cohorts and reported that compared to normal breast tissue, Polθ was the only protein significantly upregulated in both cohorts.^54^ The study indicated that Polθ overexpression could independently predict prognosis and indicate genome instability in breast cancer. Zi Wang et al. similarly reported the phenomenon of Polθ overexpression in breast cancer.^55^ Wenlong Chen et al. reported that Polθ expression was notably elevated in breast cancer tissues, and its knockdown markedly reduces the proliferation, invasion, and migration of breast cancer cells.^56^

In cervical cancer, Yuqin Zang et al. found high expression of Polθ in cancerous tissues. The suppression of Polθ markedly reduced cancer cell proliferation, invasion, and migration, highlighting its crucial role in cervical cancer development and progression.^57^

In lung cancer, Kiyoko Kawamura et al. found that high Polθ expression was observed in lung cancer samples, whereas Polθ expression was almost undetectable in paired adjacent normal lung tissues.^58^ The study revealed that patients with elevated Polθ expression had notably worse clinical outcomes than those with lower expression levels. Kazuya Shinmura et al. confirmed that Polθ was overexpressed in lung cancer, with its elevated levels associated with advanced pathological stages and a higher number of somatic mutations, suggesting its potential involvement in the initiation and progression of the disease.^59^ Xinrui Rao et al. similarly observed high expression of Polθ in lung cancer, which was significantly associated with higher tumor stage, poor prognosis, and increased tumor mutational burden.^60^ Their study further demonstrated that inhibition of Polθ suppressed lung tumorigenesis and reduced the radioresistance of lung cancer cells, and exhibited minimal toxicity toward normal lung epithelial cells. These findings revealed that Polθ represented a potential tumor-specific target, potentially useful for inhibiting lung cancer progression and enhancing radiosensitivity.

In esophageal cancer, Jian Li et al. reported that Polθ was highly expressed in esophageal squamous cell carcinoma tissues, and its expression level was negatively correlated with patient survival.^61^ Moreover, Polθ knockout significantly enhanced the sensitivity of esophageal cancer cells to multiple genotoxic drugs.

Kiyoko Kawamura et al. conducted a study on gastric cancer. They reported that Polθ expression was significantly upregulated in 11 out of 28 gastric cancer cases (39%), whereas its expression was almost undetectable in matched nontumor tissues.^58^ Elevated Polθ expression correlated with poorer clinical outcomes, suggesting its role in gastric cancer progression. Yanmei Peng et al. confirmed that Polθ positively regulated the stem cell-like properties of gastric cancer cells, and knockdown of Polθ inhibited the stemness of these cells.^62^ Simultaneously, Polθ inhibition rendered gastric cancer cells more susceptible to ferroptosis. Furthermore, the application of the Polθ inhibitor novobiocin attenuated the stemness of gastric cancer cells, increased their sensitivity to ferroptosis, and inhibited tumor growth and metastasis. Yan Wang et al. reported that high Polθ expression in patients with gastric cancer was associated with poor clinical prognosis.^63^ This study reported that Polθ knockout significantly inhibited gastric cancer cell viability and induced apoptosis. Mechanistically, Polθ knockout increased cytoplasmic and endoplasmic reticulum calcium ion concentrations, enhanced mitochondrial calcium uptake, subsequently induced mitochondrial stress and cytochrome c release, and activated the intrinsic apoptotic pathway. Furthermore, *in vivo* experiments confirmed that the Polθ inhibitor novobiocin sodium effectively suppressed tumor growth.

In colorectal cancer, Kiyoko Kawamura et al. examined 26 colon cancer samples. They found that 20 cases (77%) had substantial upregulation of Polθ expression, whereas Polθ expression was almost undetectable in paired normal tissues.^58^ The study showed that patients with elevated Polθ expression had a notably worse clinical prognosis than those with lower expression levels. The results suggested that Polθ overexpression could play a role in the progression of colorectal cancer. Similarly, Qing Yao et al. reported that Polθ expression was elevated in colorectal cancer tissues and cells, correlating with unfavorable patient outcomes.^64^ Polθ knockdown markedly suppressed colorectal cancer cell proliferation, migration, and invasion. In hereditary colorectal cancer, Ning Xu et al. identified POLQ as a key pathogenic gene.^65^ The study demonstrated that cells carrying POLQ mutations exhibited a substantial tumorigenic tendency; these mutations excessively activated the Polθ‑mediated MMEJ, leading to a high tumor mutation burden and conferring resistance to DNA‑damaging therapies. Furthermore, Ning Xu et al. effectively inhibited Polθ activity utilizing the Polθ inhibitor novobiocin, which restored tumor sensitivity to DNA‑damaging treatments, offering a potential therapeutic strategy for POLQ‑mutant drug‑resistant colorectal cancer.^65^ Screening for POLQ mutations in patients with hereditary colorectal cancer will facilitate early diagnosis and personalized treatment of this tumor type.

Qi Pan et al. conducted a study on hepatocellular carcinoma (HCC). They found that Polθ expression was significantly elevated in tumor tissues, and patients with high Polθ levels have notably shorter overall survival than those with low expression.^66^ Furthermore, Polθ knockdown effectively suppressed the growth and metastasis of hepatoma cells. Ying Cai et al. found that Polθ expression was markedly higher in HCC tissues than in normal liver tissues.^67^ Furthermore, high Polθ expression was significantly related to the progression of T stage and pathological stage in patients with HCC, and high Polθ expression was closely associated with reduced survival in these patients. In cellular models, Polθ reduction inhibited HCC proliferation, whereas Polθ overexpression enhanced hepatoma cell proliferation. These findings provided new perspectives for the individualized treatment of HCC. Similarly, Junnan Li et al. found that Polθ expression was notably higher in HCC tissues than in normal tissues, and elevated levels of Polθ were associated with reduced overall survival.^68^

Agnieszka Smolinska et al. conducted research on pancreatic cancer and reported that Polθ expression was significantly elevated in tumor tissues.^69^ Polθ deficiency impeded pancreatic cancer initiation and progression, and the overall survival of experimental mice was significantly prolonged following Polθ loss.^69^ Laura Del Puerto-Nevado et al. reported that Polθ expression was elevated in pancreatic cancer and correlated with reduced patient survival, suggesting its role as an independent prognostic factor.^70^

In thyroid cancer, C. Corbin Frye et al. analyzed Polθ expression in 513 papillary thyroid carcinoma samples and 59 normal thyroid tissue samples.^71^ The study revealed significantly elevated Polθ expression in papillary thyroid carcinoma tissues compared to normal thyroid tissues. High Polθ expression was notably linked to thyroid cancer dedifferentiation and reduced progression-free intervals. Additionally, *ex vivo* experiments revealed that application of a Polθ inhibitor markedly inhibited the growth of thyroid cancer. The results suggest that elevated Polθ expression in thyroid cancer tissues could be an important clinical biomarker and a potential target for therapy.

Han Bai et al. conducted a study on salivary adenoid cystic carcinoma and reported that Polθ overexpression promoted salivary adenoid cystic carcinoma progression, and high Polθ expression was closely associated with poor prognosis in patients.^72^

Sahiti Chukkapalli and colleagues conducted a study on neuroblastoma and reported that Polθ was overexpressed in cancer cells and tumor tissues, and Polθ overexpression predicted poor survival outcomes in neuroblastoma patients.^73^ The study demonstrated that Polθ knockout significantly inhibited cancer cell proliferation and enhanced sensitivity to DNA-damaging agents, signifying that Polθ was a potentially viable therapeutic target in neuroblastoma.

Qun Li and colleagues found that Polθ expression was notably higher in multiple myeloma patients than in healthy individuals, with its overexpression linked to advanced disease stages and poor prognosis.^74^ Furthermore, Polθ inhibition suppressed the growth of multiple myeloma. Moreover, application of a Polθ inhibitor sensitized multiple myeloma cells to melphalan. These results indicated that Polθ may serve as a promising prognostic biomarker in multiple myeloma, and combining a Polθ inhibitor with melphalan represented a potential therapeutic strategy for multiple myeloma.

Umeshkumar Vekariya and colleagues demonstrated that Polθ expression was significantly elevated in leukemia cells relative to normal cells and demonstrated that Polθ was implicated in leukemogenesis.^75^ Further experiments in that study revealed that knocking out Polθ significantly inhibited cancer cell growth.

In prostate cancer, Chia-Hao Kuei et al. found that Polθ expression levels were significantly elevated in prostate cancer tissues compared to healthy tissues.^76^ Polθ upregulation was common in metastatic castration-resistant prostate cancer and indicated poor patient prognosis. Polθ knockdown markedly enhanced tumor cell sensitivity to docetaxel. Febina Ravindran et al. conducted whole-exome sequencing on 58 primary prostate tumor samples and further suggested that Polθ may represent a viable therapeutic target in prostate cancer.^77^

Polθ is significantly linked to the initiation and development of multiple cancers. By promoting genomic instability and facilitating mutation accumulation, Polθ contributes to tumorigenesis and disease progression, thereby enhancing tumor proliferation, migration, invasion, and the maintenance of cancer stem cell stemness. These mechanisms clarify why patients with elevated Polθ expression have significantly worse survival outcomes than those with lower expression levels.

## 4. Regulation of Polθ and MMEJ

The impact of Polθ on tumorigenesis and development has been increasingly reported, leading to increased interest in the regulatory mechanisms of Polθ and MMEJ. Simultaneously, research on the regulation of Polθ expression within oncology has increased (Table 2). These studies have advanced the understanding of Polθ's role in tumorigenesis and development, providing essential support for future fundamental research and clinical applications.

**Table 2.** The regulation of Polθ and MMEJ by proteins and pathways.

Proteins and pathways that regulated Polθ and MMEJ	Publication time	Regulation of Polθ and MMEJ	Reference (PMID)
HR pathway	2015	Polθ expression and MMEJ activity increased when HR was deficient	PMID 25642960; PMID 25642963
NHEJ pathway	2022	Polθ expression and MMEJ activity increased when NHEJ was inhibited	PMID 35972384
MSH2-MSH3 complex	2023	MSH2-MSH3 complex inhibited MMEJ	PMID 37140056
MLH1	2025	Polθ expression level was elevated following MLH1 knockout	PMID 41163511
Cyclin D1	2025	Cyclin D1 upregulated the expression of Polθ, thereby driving MMEJ	PMID 40608419
EWSR1	2025	The loss of EWSR1 reduced Polθ expression and impaired MMEJ	PMID 40568108
EWS-FLI1	2025	EWS-FLI1 inhibited MMEJ	PMID 40568108
ZEB1	2020	ZEB1 inhibited Polθ expression, thereby suppressing MMEJ	PMID 33239429
S6K	2024	S6K reduces Polθ expression level	PMID 38925361
FTO	2024	FTO increased Polθ expression level	PMID 38270643
ALDH1A1	2023	ALDH1A1 enhanced Polθ expression	PMID 37429899
APOBEC3A	2025	APOBEC3A promoted MMEJ	PMID 41278855
Polλ	2022	Polλ promoted the MMEJ process	PMID 36536104
FANCD2	2016	FANCD2 enhancing MMEJ capacity	PMID 27264184
PLK1	2023	PLK1 phosphorylated Polθ to promote MMEJ	PMID 37674080
RHINO	2023	Phosphorylated RHINO interacted with Polθ to enable MMEJ repair	PMID 37440612
CIP2A-TOPBP1 complex	2025	CIP2A–TOPBP1 complex enhanced MMEJ activity	PMID 41309571

### 4.1. Regulation of Polθ and MMEJ by DNA DSB repair pathways

Regarding the regulatory mechanisms of Polθ expression, current research has predominantly focused on its regulation by the DDR system. In cells, DSBs are primarily repaired through three major pathways: HR, NHEJ, and MMEJ.^17^ Among these, MMEJ functions as an alternative and inefficient DSB repair pathway. This inefficiency arises from two main factors: the repair mechanism is significantly error-prone, causing cells to deprioritize this pathway for DDR; HR and NHEJ actively inhibit MMEJ during the repair process.^24^ ^,^ ^78-80^ When tumor cells exhibit deficiencies in HR and NHEJ pathways, they are compelled to rely on alternative repair mechanisms to compensate for DSB repair.^40^ Under such circumstances, Polθ expression levels and MMEJ activity become abnormally elevated. As early as 2015, two independent research teams simultaneously reported that cells lacking HR repair capacity became dependent on MMEJ for compensatory repair.^81^ ^,^ ^82^ Moreover, Jeffrey Patterson-Fortin et al. found that inhibition of NHEJ repair capacity significantly increased Polθ expression and MMEJ activity.^83^

### 4.2. Regulation of Polθ and MMEJ by other DDR pathways

Recent studies indicated that proteins in the MMR pathway could alter Polθ expression and MMEJ activity. Research by Jung-Min Oh and Bac Viet Le revealed that the MMR complex, comprising MutS protein homologs 2 (MSH2) and MutS protein homologs 3 (MSH3), interacted with the chromatin remodeling protein SMARCAD1 and was recruited to DSB sites.^84^ ^,^ ^85^ At this point, the MSH2-MSH3 complex inhibited MMEJ by preventing the recruitment of Polθ to the damage site. In 2025, our team's findings indicated that knockout of the MMR protein MutL homolog 1 (MLH1) significantly impaired DSB repair capacity in esophageal cancer cells, with HR and NHEJ repair capabilities significantly reduced.^86^ Simultaneously, Polθ expression levels were significantly elevated after MLH1 knockout. This indicated that MLH1 loss significantly increased the utilization of the MMEJ pathway to compensate for DSB repair.^86^ Furthermore, to investigate whether defects in other pathways of the DDR system led to dependency on Polθ, Wanjuan Feng et al. predicted through CRISPR-based genetic screening that protein loss from other repair pathways would confer Polθ dependency.^87^ However, the specific mechanisms underlying this prediction and its reliability require additional validation and clarification in future research.

### 4.3. Regulation of Polθ and MMEJ by proteins

Recent studies indicated that in addition to the DDR system's regulatory role, various proteins and signaling pathways also influenced Polθ expression and MMEJ. Cyclin D1 is a common oncoprotein that is overexpressed in various tumors. Jithma *P*. Abeykoon and colleagues reported that cyclin D1 was overexpressed in mantle cell lymphoma, and this overexpression increased DNA damage and specifically upregulated MMEJ activity.^88^ Moreover, cyclin D1 activated POLQ transcription and drove MMEJ. Shuhei Asada et al. revealed that the splicing factor Ewing sarcoma R1 (EWSR1) was essential for the precise splicing of POLQ pre-mRNA, which was vital for the proper expression of the POLQ gene.^89^ The loss of EWSR1 reduced Polθ expression and impaired MMEJ repair capacity. Simultaneously, this study demonstrated that Ewing sarcoma-FLI1 (EWS-FLI1) expression resulted in defective MMEJ repair, whereas EWS-FLI1 knockdown significantly restored MMEJ repair capacity. Zinc finger E-box binding homeobox 1 (ZEB1) is a transcription factor that triggers epithelial-to-mesenchymal transition. Melanie K. Prodhomme et al. confirmed that ZEB1 directly interacted with the POLQ promoter and inhibited POLQ expression, suppressing MMEJ repair capacity in breast cancer.^90^ ^,^ ^91^

Various downstream effector molecules and enzymes of signaling pathways can participate in the regulation of Polθ expression. Ribosomal protein S6 kinase (S6K) was overexpressed in prostate tumors and aids in tumor cell resistance to radiation.^92^ Alison Clark et al. confirmed that the absence of S6K decreased the expression of nuclear factor kappa B (NF-κB), sequestosome-1 (SQSTM1), and Polθ.^92^ Moreover, pharmacological inhibition of S6K downregulated NF-κB/p62 signaling and significantly reduced Polθ expression. The fat mass and obesity-associated protein (FTO) is linked to the advancement of multiple cancers. Yichen He et al. reported that FTO regulated m6A modification to influence POLQ expression, thereby promoting cell proliferation in clear cell renal cell carcinoma.^93^ Other studies demonstrated that aldehyde dehydrogenase 1A1 (ALDH1A1) could be involved in regulating Polθ expression. Kousalya Lavudi et al. reported that ALDH1A1 could participate in POLQ transcriptional activation by activating the retinoic acid signaling pathway, enhancing Polθ expression levels in ovarian cancer cells.^94^ This offered a novel explanation for the molecular mechanism underlying Polθ overexpression in ovarian cancer.

Additionally, some proteins can directly affect MMEJ activity without affecting Polθ expression. The APOBEC3 cytidine deaminase family is linked to the development and advancement of multiple cancers. APOBEC3A, a member of the APOBEC3 family, competed with RPA for ssDNA overhangs, exposing microhomology sequences and redirecting DNA DSB repair toward the error-prone MMEJ pathway.^95^ Moreover, Gurushankar Chandramouly et al. reported that MMEJ activity was not solely mediated by Polθ; Polλ could participate in and promote the MMEJ process.^96^ This finding clarified a distinct Pol *λ*-dependent MMEJ mechanism, enriching the understanding of the regulatory network of the MMEJ pathway. Zeina Kais et al. demonstrated that Fanconi anemia complementation group D2 (FANCD2) aided in recruiting Polθ to DNA damage sites, thereby enhancing MMEJ capacity.^97^

The repair pathways for DSBs exhibit cell cycle dependence. During interphase, DSBs are primarily repaired through NHEJ and HR, which are completely suppressed during mitosis. In 2023, Camille Gelot et al. reported that Polθ specifically repaired DSBs in mitosis, maintaining genomic integrity.^35^ The study demonstrated that Polo-like kinase 1 (PLK1) phosphorylated and activated Polθ function during mitosis. During mitosis, phosphorylated Polθ was recruited to double-strand break sites to facilitate end-joining repair.^35^ Simultaneously, Alessandra Brambati et al. confirmed that similar findings, showing that RAD1-interacting nuclear orphan protein 1 (RHINO) accumulated during mitosis and was phosphorylated by PLK1.^36^ The modified RHINO interacted with Polθ, enabling its recruitment to DSB sites to facilitate subsequent MMEJ. Moreover, Peter R. Martin et al. offered additional insight into the regulatory mechanisms of MMEJ during mitosis.^98^ They demonstrated that the CIP2A-TOPBP1 complex enhanced MMEJ activity by facilitating the efficient localization of Polθ during mitosis, contributing to DSB repair.

## 5. Development and clinical translation of Polθ inhibitors

In 2015, two research teams simultaneously reported that tumor cells deficient in HR relied on MMEJ.^81^ ^,^ ^82^ This finding propelled Polθ into an emerging therapeutic target for cancer treatment. As the functions and underlying molecular mechanisms of Polθ have been continuously clarified, the understanding of Polθ and MMEJ has deepened. Therefore, the potential of Polθ in cancer therapy has become increasingly prominent, solidifying its status as a highly attractive therapeutic target. Since the first Polθ inhibitor was reported in 2021,^99^ research interest in this field has significantly expanded. As Polθ inhibitors have become a research hotspot in cancer therapy, many new compounds are constantly emerging. Over the past 5 y, an increasing number of patents on inhibitors targeting Polθ have been published.^100^ For the helicase domain, there were 37 patents published. Additionally, 45 patents for inhibiting the Polθ polymerase domain were published. Recently, several specific Polθ-targeting inhibitors have been developed (Table 3), with some already advancing to clinical trial stages. These Polθ inhibitors are undergoing active preclinical and clinical investigations. Future results from these studies are anticipated to accelerate the clinical translation of Polθ inhibitors, offering novel directions for cancer therapy.

**Table 3.** Summary of Polθ inhibitors. IC50, half-maximal inhibitory concentration; MMEJ, microhomology-mediated end joining.

Inhibitor name	Publication time	The target of the inhibitor	Chemical structures	IC50	Reference (PMID)
ART558	2021	Polymerase domain of Polθ	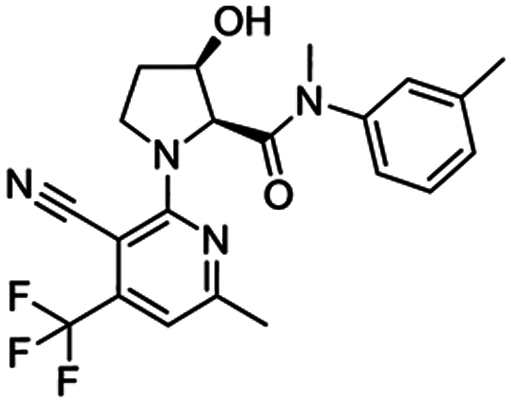	7.9 nM (Inhibition of Polθ polymerase)	PMID 34140467
Novobiocin	2021	Helicase domain of Polθ	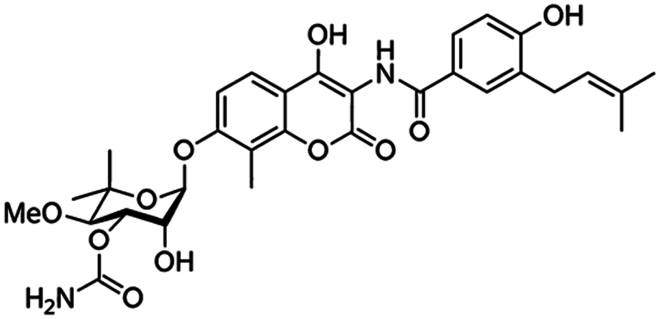	24 μM (Inhibition of Polθ helicase)	PMID 34179826
RP-6685	2022	Polymerase domain of Polθ	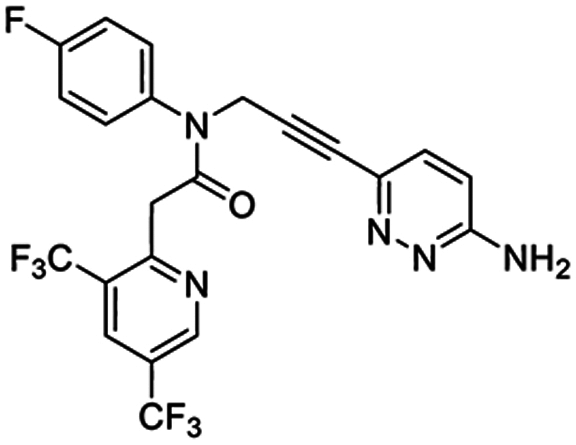	0.55 nM (Inhibition of Polθ polymerase)	PMID 36126059
ART899	2023	Polymerase domain of Polθ	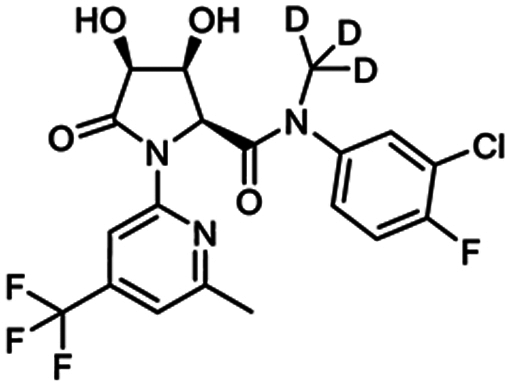	180 nM (Inhibition of MMEJ)	PMID 36689546
25 d	2024	PARP1 and helicase domain of Polθ	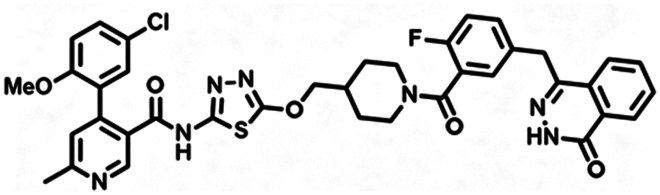	46 nM (Inhibition of Polθ helicase)	PMID 38375763
RTx-161	2024	Polymerase domain of Polθ	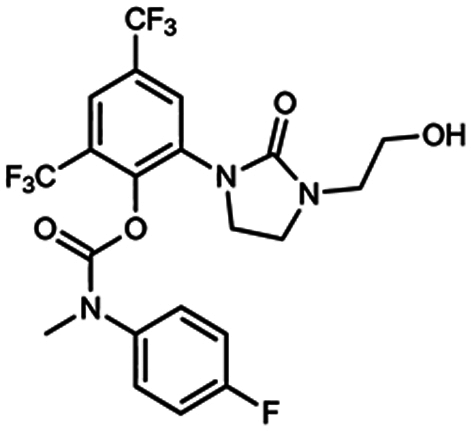	4.1 nM (Inhibition of Polθ polymerase)	PMID 38580648
RP-2119	2025	Helicase domain of Polθ	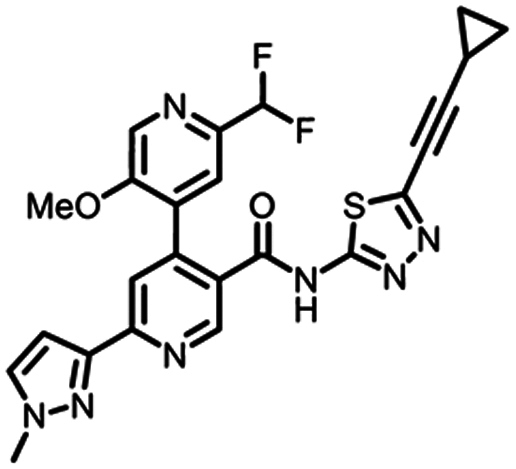	0.7 nM (Inhibition of Polθ helicase)	PMID 40920169
RTx-303	2025	Polymerase domain of Polθ	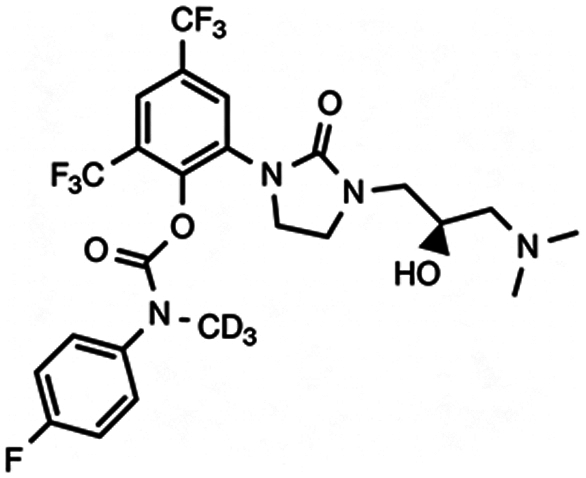	5.1 nM (Inhibition of Polθ polymerase)	PMID 41124685
Compound 33	2025	Polymerase domain of Polθ	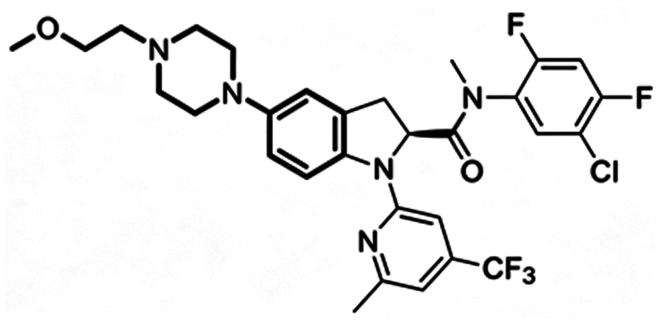	9.6 nM (Inhibition of Polθ polymerase)	PMID 41213161
VS-13	2026	Polymerase domain of Polθ	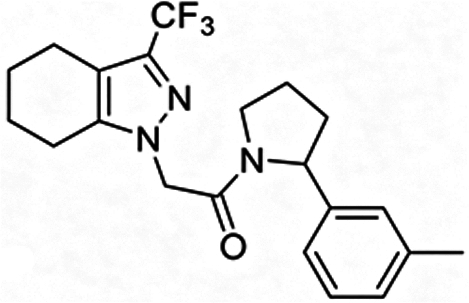	1.47 μM (Inhibition of Polθ polymerase)	PMID 41459625
SY-589	2026	Helicase domain of Polθ	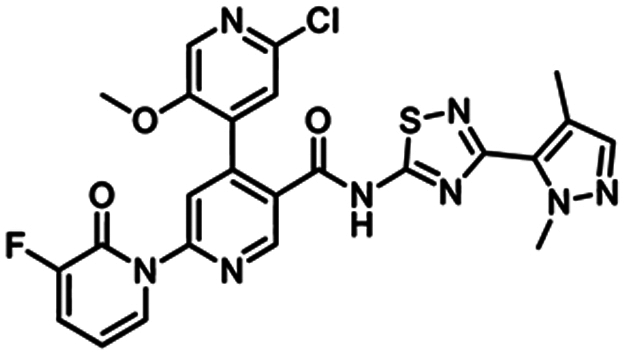	2.29 nM (Inhibition of Polθ helicase)	PMID 41575169
Compound 20	2026	Polymerase domain of Polθ	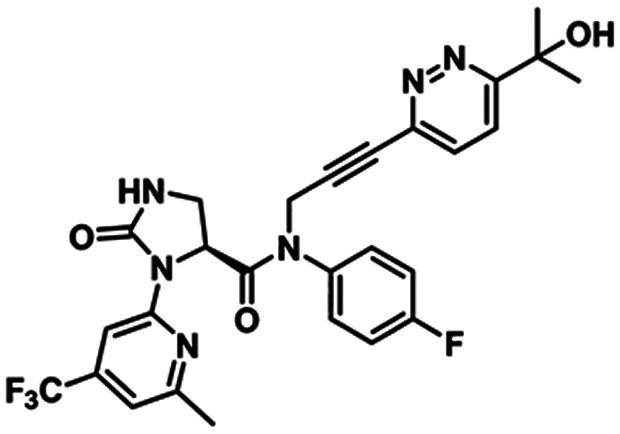	1.3 nM (Inhibition of Polθ polymerase)	PMID 41611653
XL-20	2026	Helicase domain of Polθ	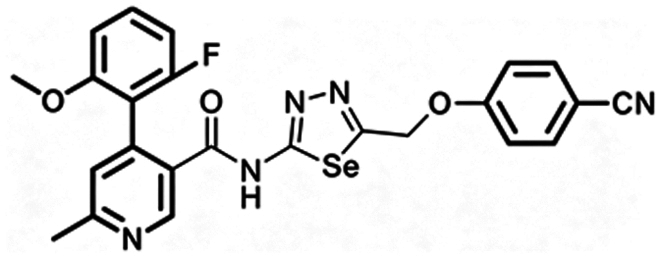	4.3 nM (Inhibition of Polθ helicase)	PMID 42066378

Polθ is a multifunctional protein possessing dual enzymatic activities: helicase and polymerase.^28^ Both activities are crucial for MMEJ. Current Polθ inhibitors are categorized into two main classes based on their structural and functional traits, focusing on either the polymerase or helicase domain of Polθ. This offers distinct strategic avenues for the precise modulation of Polθ function and intervention in tumor progression.

### 5.1. Inhibitors targeting the helicase domain of Polθ

Novobiocin, a coumarin antibiotic from Streptomyces, treats bacterial infections by targeting the ATP-binding site of DNA gyrase B.^101^ In 2021, large-scale drug screening and experimental validation revealed that novobiocin specifically binds to the helicase domain of Polθ, inhibiting MMEJ repair.^99^ ^,^ ^102^ ^,^ ^103^ The study utilized CRISPR-Cas9 technology to knock out Polθ in cells, demonstrating that Polθ-knockout cells exhibited greater resistance to novobiocin relative to wild-type cells. These findings confirmed that novobiocin specifically targets Polθ in human cells. Moreover, the study revealed that elevated Polθ levels may predict tumor cell sensitivity to novobiocin.

Philippe Mochirian et al.^104^ reported that RP-2119 was a selective, potent, and bioavailable inhibitor of the Polθ helicase. The study showed that RP-2119 significantly enhanced the antitumor effects of the PARP inhibitor olaparib in HR-deficient (HRD) cell lines and patient-derived xenograft models, without increasing hematological toxicity. Collectively, these results suggested a novel potential strategy for combination therapy in HR‑deficient tumors.

A potent dual Polθ/PARP inhibitor, 25 d, was developed by Luyu Ma et al. They demonstrated that 25 d exhibited strong inhibitory effects on the helicase activity of Polθ and PARP1 at low nanomolar concentrations.^105^ The study showed that 25 d caused DNA damage and apoptosis, leading to significant antitumor effects in MDA-MB-436 cells and xenograft models. Overall, 25 d was a dual Polθ/PARP inhibitor and exhibited promising potential for the treatment of HRD tumors.

SY-589 was identified as a potent, selective, and orally bioavailable Polθ helicase inhibitor and showed strong antitumor efficacy *ex vivo* in HRD tumors.^106^ SY-589 demonstrated significant synergy with the PARP inhibitor olaparib both *ex vivo* and *in vivo*, markedly enhancing antitumor effects. Overall, these findings position SY-589 as a promising Polθ inhibitor candidate, providing a new therapeutic option for cancer treatment.

In 2026, Luyu Ma et al. discovered that compound XL-20 was a potent and highly orally bioavailable Polθ ATPase inhibitor.^107^ This inhibitor had strong inhibitory effects on Polθ ATPase at low nanomolar concentrations, and showed synergistic antitumor effects in HRD cells and xenograft models when combined with PARP inhibition. Notably, XL-20 significantly activated the cGAS-STING pathway and upregulated PD-L1 expression, indicating its potential for combined use with immunotherapy. In summary, XL-20 had a dual mechanism of action and high oral bioavailability, making it a promising inhibitor with great potential for development.

### 5.2. Inhibitors targeting the Polθ polymerase domain

To target Polθ in cancer, ART558, a Polθ inhibitor characterized by high potency, selectivity, and low molecular weight, was developed. ART558 inhibited the function of the Polθ protein by binding to its polymerase domain.^108^ Similar to novobiocin, ART558 was identified through extensive drug screening and subsequently validated as a specific Polθ inhibitor. However, its mechanism of action differed from that of novobiocin. Novobiocin and ART558 inhibited Polθ by binding to its helicase and polymerase domains, respectively.^108^ ^,^ ^109^ Polθ inhibition by ART558 was highly specific. Even at elevated concentrations, it did not inhibit other polymerases, such as Polα and Polγ, or kinases, including PARP1 and PARP2. Gonzalo Rodriguez-Berriguete et al. further confirmed that ART558 was an inhibitor specifically targeting the DNA polymerase domain of Polθ.^109^ Furthermore, the study reported another potent and specific Polθ polymerase inhibitor, ART899, which exhibited superior metabolic stability compared to ART558. *In vivo* studies demonstrated that ART899-mediated inhibition of Polθ, in combination with fractionated radiotherapy, was well-tolerated and significantly enhanced tumor growth inhibition compared to radiotherapy alone. The results strongly supported the clinical exploration of combining Polθ inhibitors with radiotherapy.

RP-6685, a selective and orally bioavailable Polθ inhibitor, was identified and synthesized by Bubenik et al.^110^ RP-6685 and ART558 both inhibited Polθ by binding to the polymerase domain of Polθ. *In vivo* and *ex vivo* studies demonstrated that cells deficient in HR repair were sensitive to RP-6685. Currently, research on RP-6685 as a novel Polθ inhibitor remains relatively limited. To achieve clinical application, additional preclinical studies and subsequent clinical trials are required to confirm its safety and efficacy. Furthermore, Wenlin Fan and colleagues identified VS-13 as a Polθ polymerase inhibitor through virtual screening.^111^ VS-13 exhibited favorable antiproliferative activity against BRCA2-deficient tumor cells.

RTx-303, a small-molecule inhibitor of Polθ polymerase, was developed with an oral bioavailability of 88%.^112^ RTx-303 effectively inhibited Polθ polymerase activity and demonstrated a favorable tolerability profile, supporting its potential for further clinical development and application. Similarly, Polθ polymerase inhibitor RTx-161 selectively eliminated HRD cells and exhibited synergistic antitumor effects with PARP inhibitors, further expanding the combination therapy strategies involving Polθ inhibitors.^113^

Jinyang Zhang et al. detailed the identification and enhancement of new Polθ inhibitors.^114^ These inhibitors possessed an aryl alkyne core scaffold that extended into the peripheral channel of the Polθ polymerase domain, thereby enhancing target binding affinity. Compound 20 showed a strong inhibitory effect on Polθ polymerase and significant antiproliferative activity in HRD cancer cell lines among Polθ inhibitors.^114^ Moreover, compound 20 exhibited high oral bioavailability in mice and rats. *In vivo* xenograft model studies showed that compound 20 effectively suppressed tumor growth without causing noticeable toxicity. These findings underscored the promise of the aryl alkyne scaffold in creating orally active Polθ inhibitors and offered crucial insights for future structural design and optimization of these inhibitors.

Ziheng Yu et al. reported that a series of Polθ polymerase inhibitors were designed and optimized, enhancing potency and pharmacokinetic properties by targeting a previously uncharacterized binding pocket.^115^ Among them, compound 33 exhibited significant Polθ inhibitory activity and potent antiproliferative effects in HR‑deficient cancer cells.^115^ In the MDA-MB-436 xenograft model, combining compound 33 with the PARP inhibitor olaparib significantly increased DNA damage accumulation and notably inhibited tumor growth. Pharmacokinetic analyzes demonstrated that compound 33 showed favorable dose-dependent properties, while safety assessments revealed good tolerability. These findings identified compound 33 as a potent Polθ inhibitor.

### 5.3. Clinical translation of Polθ inhibitors

Polθ inhibitors represent a promising therapeutic approach in oncology. Following the successful development and optimization of multiple Polθ inhibitors, several candidates have advanced into clinical trials (Table 4), facilitating the translation of Polθ-targeted strategies from fundamental research to clinical application. Specifically, clinical trials across various stages are being conducted in order.

**Table 4.** Summary of clinical trials related to Polθ inhibitors.

Clinical trial ID	Start date	Agent	Phase	Indication	Combination	Current status
NCT04991480	2021	ART4215	I/II	Advanced solid tumors, Metastatic solid tumors	Monotherapy, Talazoparib, Niraparib	Completed
NCT05687110	2023	Novobiocin	I	Solid tumors	Monotherapy	Recruiting
NCT05898399	2023	ART6043	I/II	Advanced solid tumors, Metastatic solid tumors	Monotherapy, Olaparib	Active, not recruiting
NCT06077877	2023	GSK4524101	I/II	Solid tumors	Monotherapy, Niraparib	Active, not recruiting
NCT06545942	2024	MOMA-313	I	Advanced solid tumors, Relapsed solid tumors, Metastatic solid tumors	Monotherapy, Olaparib	Recruiting
NCT06666270	2024	SYN818	I	Advanced solid tumors, Metastatic solid tumors	Monotherapy	Recruiting
NCT06686745	2024	SIM0508	I	Advanced solid tumors, Metastatic solid tumors	Monotherapy, Olaparib	Recruiting
NCT06998173	2025	DAT-1604	I	Advanced solid tumor	Monotherapy	Not yet recruiting

The Phase I/II clinical trial (NCT04991480), launched in 2021, sought to assess the safety, tolerability, and efficacy of ART4215 both as a standalone treatment and in conjunction with PARP inhibitors. In 2023, multiple clinical trials were conducted simultaneously. A Phase I trial (NCT05687110) was designed to assess the safety and optimal dosing of novobiocin in patients with solid tumors harboring DNA repair gene alterations. The Phase I/II trial (NCT05898399) assessed the safety, tolerability, and efficacy of ART6043, both as a standalone treatment and in combination with olaparib, for advanced or metastatic solid tumors. The Phase I/II trial (NCT06077877) evaluated the maximum tolerated dose and therapeutic efficacy of GSK4524101, both as a monotherapy and in combination with niraparib, in patients with solid tumors.

Further trials advanced in 2024. A Phase I clinical trial (NCT06545942) aimed at advanced metastatic solid tumors with HR deficiency assessed the safety, tolerability, and efficacy of MOMA-313 both as a monotherapy and in combination with a PARP inhibitor, establishing a basis for further clinical development. A Phase I trial (NCT06666270) was initiated to evaluate the safety, tolerability, pharmacokinetics, pharmacodynamics, and antitumor efficacy of SYN818 in patients with advanced solid tumors. The Phase I clinical trial (NCT06686745) assessed the safety, tolerability, and efficacy of SIM 0508, both as a monotherapy and in combination with olaparib, in patients with advanced or metastatic solid tumors. A Phase I clinical trial (NCT06998173) commenced in 2025 to assess the safety, tolerability, and efficacy of DAT-1604 as a monotherapy for advanced solid tumors. Collectively, these ongoing clinical trials are expected to substantially influence the future direction of Polθ-targeted therapy. Their results are expected to significantly influence the research direction in Polθ inhibitors. Positive outcomes would greatly accelerate the clinical translation of Polθ inhibitors in cancer therapy, offering new treatment options for cancer patients.

Preclinical and clinical studies showed that Polθ inhibitor monotherapy exerted limited anti-tumor activity, while its combination with PARP inhibitors produced prominent synergistic effects in DNA repair-deficient cancers.^108^ ^,^ ^112^ ^,^ ^116^ ^,^ ^117^ The limited therapeutic efficacy of single-agent Polθ inhibition stems largely from the functional redundancy and compensatory capacity of DNA repair networks. Monotherapy with Polθ inhibitors exclusively suppresses the MMEJ pathway. In this context, cancer cells can still resolve endogenous and replication stress-induced DSBs via HR and NHEJ pathways, maintaining genomic stability and evading Polθ inhibition-mediated cell death.

A combination of Polθ inhibitors and PARP inhibitors elicits potent synthetic lethality by disrupting complementary DNA repair pathways. PARP proteins are critical for base excision repair and single-strand break repair.^15^ PARP inhibitors cause persistent single-strand break accumulation that converts into cytotoxic DSBs during DNA replication.^118^ For HRD cancer cells, HR repair is impaired, making these cells exclusively dependent on MMEJ to resolve lethal DSB lesions and sustain genomic integrity. Dual blockade of PARP and Polθ completely abolishes two essential repair cascades in HRD cancer cells: Polθ-mediated MMEJ DSB repair and PARP-dependent base excision repair and single-strand break repair. The simultaneous disruption of core and backup DNA repair pathways in HRD cancer cells leads to irreversible DSB accumulation and widespread replication fork collapse, which ultimately induces cell cycle arrest and robust cancer cell death.

In summary, the functional redundancy of DNA repair pathways is the primary cause of the limited clinical efficacy of Polθ inhibitor monotherapy. The synthetic lethal interplay between PARP-dependent DDR and Polθ-mediated DDR establishes a solid mechanistic rationale for their combined application. This combinatorial strategy effectively bypasses tumor compensatory repair resistance and markedly improves antitumor therapeutic outcomes. The combined treatment of Polθ inhibitors and PARP inhibitors for cancers may be a direction for the future application of Polθ inhibitors.

### 5.4. Comparative analysis of Polθ polymerase inhibitors and helicase inhibitors

In the mechanism of action, Polθ polymerase inhibitors occupy the catalytic active pocket of Polθ-PolD, competitively blocking primer-template DNA binding, and directly abolish the strand extension step of MMEJ.^26^ ^,^ ^100^ Their inhibition is restricted to the polymerase catalytic function and does not interfere with the helicase-mediated microhomology search process. In contrast, Polθ helicase inhibitors target the ATPase motif of Polθ-HelD, suppressing ATP-dependent ssDNA scanning, and exerting an upstream blockade of the entire MMEJ repair.^26^ ^,^ ^100^

In target selectivity, Polθ polymerase inhibitors face prominent selectivity challenges: human cells contain multiple homologous polymerases (Polα, Polδ, Polε). Many early Polθ polymerase inhibitors cross-inhibit polymerases, inducing replication stress in normal cells.^26^ Optimized derivatives show improved Polθ preference but still retain weak activity against other polymerases. Polθ helicase inhibitors display fewer homologous human helicase off-targets. Overall, Polθ helicase inhibitors exhibit superior target selectivity relative to first-generation Polθ polymerase inhibitors.

In pharmacokinetic properties, most Polθ polymerase inhibitors are small polar molecules with cell permeability, but hepatic metabolic clearance limits sustained intratumoral drug exposure. Many analogs require high administration doses to achieve effective MMEJ suppression *in vivo*. Polθ helicase inhibitors generally possess lipophilicity, with longer half-lives in plasma and tumor tissues. However, excessive lipophilicity leads to elevated nonspecific tissue accumulation, which exacerbates systemic exposure risk.^26^

In preclinical toxicity, both two inhibitor classes trigger on-target toxicity derived from Polθ's indispensable physiological roles. But the two inhibitor classes do not cause severe acute organ toxicity at effective therapeutic concentrations.

In translational development progress, both Polθ polymerase inhibitors and helicase inhibitors have ongoing clinical trials for clinical translation.^100^ The effect of Polθ inhibitors alone in the treatment of tumors is limited, while the effect of Polθ inhibitors combined with PARP inhibitors is obvious. At present, the combination of Polθ inhibitors and PARP inhibitors has become the mainstream direction in clinical translation.^108^ ^,^ ^112^ ^,^ ^113^ In addition, the design of dual Polθ/PARP inhibitors to simultaneously inhibit Polθ and PARP to kill tumors is also a novel idea for clinical translation. Luyu Ma et al. reported a dual Polθ/PARP inhibitor 25 d, which had significant advantages.^105^ At low nanomolar concentrations, 25 d showed strong inhibitory effects on both Polθ and PARP1. Compared with the combination of Polθ inhibitors and PARP inhibitors, 25 d treatment showed a stronger antitumor effect. Importantly, tumors with acquired PARP inhibitor resistance were sensitive to 25 d. While 25 d displayed obvious antitumor effects, further research is still needed to enhance its selectivity and pharmacokinetic property.

## 6. Synthetic lethal strategies targeting Polθ in cancer

### 6.1. Concept and application of synthetic lethality

**Figure 3.**
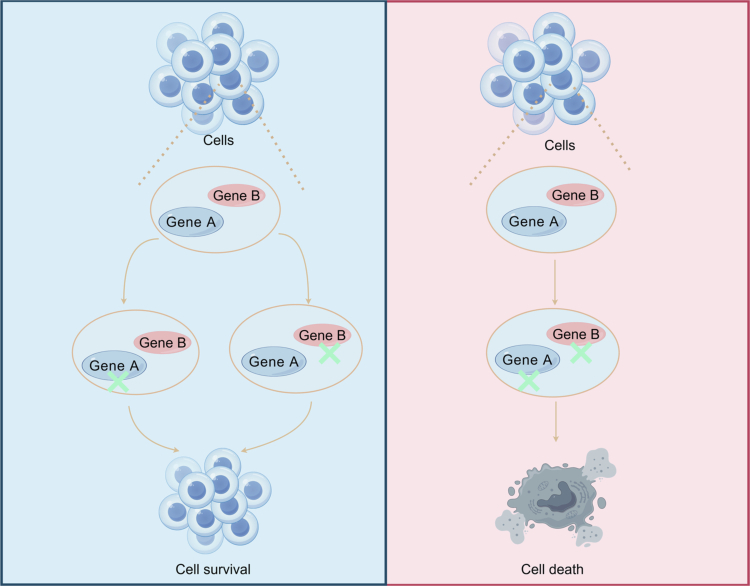
The concept model of synthetic lethality. Synthetic lethality is a genetic interaction where loss-of-function of two genes together causes cell death, whereas inactivation of either gene alone remains compatible with cell survival.

Synthetic lethality, initially observed in Drosophila, described a genetic interaction where the inactivation of either of two genes individually allowed cell survival, but their simultaneous inactivation led to cell death (Figure 3).^43^ ^,^ ^119^ In 2005, two separate studies showed that PARP inhibitors induced cell death in cancer cells lacking breast cancer susceptibility gene 1/2 (BRCA1/2).^120^ ^,^ ^121^ This discovery established a landmark example of synthetic lethality strategies in cancer. The subsequent clinical application of PARP inhibitors in patients with BRCA1/2-deficient tumors substantially aroused interest in synthetic lethal therapeutic strategies in cancer and stimulated the identification of more such strategies.^118^ ^,^ ^122-124^ The implementation of synthetic lethal strategies in cancer largely relies on the compensatory interactions among DDR pathways. Cancer cells with deficiencies in a DNA repair pathway often rely on alternative pathways to sustain repair and ensure survival.^125^ ^,^ ^126^ Based on the concept of synthetic lethality, inhibiting the alternative repair pathway in cells with defective DSB repair can selectively kill tumor cells while sparing normal cells.^127^

**Figure 4.**
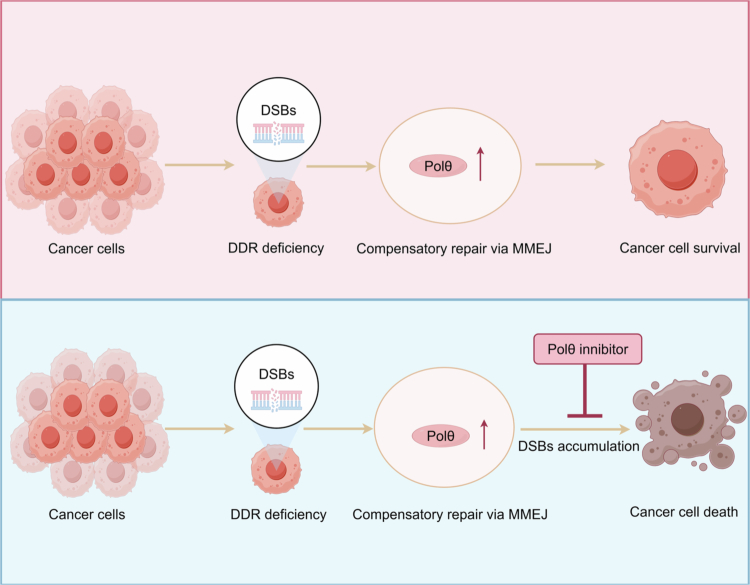
The principle of synthetic lethality targeting Polθ. Cancer cells with DDR defects rely on MMEJ repair pathways to compensate for repair and maintain cell survival. Inhibiting MMEJ repair pathway through Polθ inhibitors can lead to the accumulation of DNA damage and the death of cancer cells with DDR defects. DSBs, double-strand breaks; DDR, DNA damage repair; Polθ, DNA polymerase theta; MMEJ, microhomology-mediated end joining.

Multiple DDR pathways exist in cells to collectively maintain genomic stability. In tumor cells, deficiencies in HR and NHEJ are common. Under such conditions, cancer cells rely on alternative repair mechanisms to compensate for DDR.^125^ ^,^ ^128^ MMEJ represents a critical alternative DDR pathway. When HR or NHEJ are defective, tumor cells increasingly depend on MMEJ to repair DNA damage.^124^ ^,^ ^129^ The principle of synthetic lethality underpins the use of Polθ inhibitors to selectively target and kill tumor cells, forming the theoretical basis for Polθ-targeted cancer therapies (Figure 4).^130^

### 6.2. Synthetic lethality between Polθ and HR

**Figure 5.**
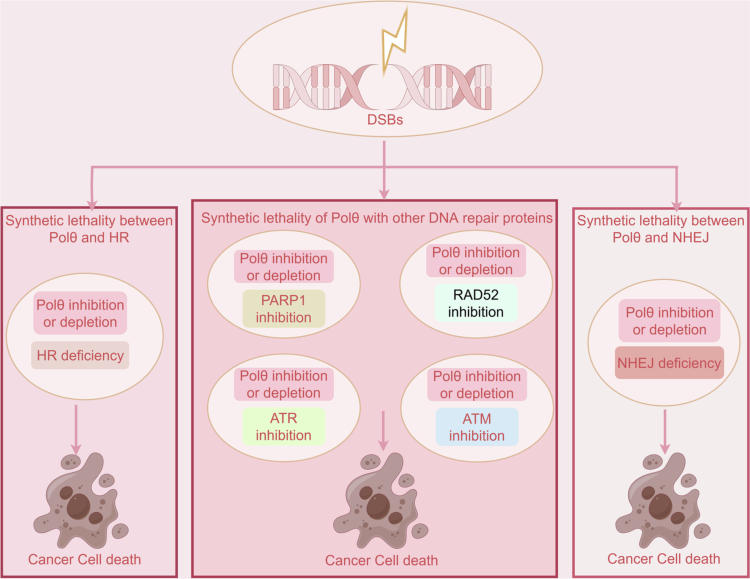
Synthetic lethal partners with Polθ inhibition or depletion. Polθ inhibition or depletion can cause the death of cancer cells with HR deficiency or NHEJ deficiency. The application of synthetic lethality between Polθ and DNA repair proteins (PARP1, RAD52, ATM, and ATR) can lead to cancer cells death. DSBs, double-strand breaks; Polθ, DNA polymerase theta; HR, homologous recombination; NHEJ, nonhomologous end-joining; PARP1, poly (ADP-ribose) polymerase 1; ATM, ataxia-telangiectasia mutated serine/threonine kinase; ATR, ATM and RAD3-related serine/threonine kinase.

In 2015, two research groups independently identified a synthetic lethal interaction between HR deficiency and Polθ in tumors.^81^ ^,^ ^82^ This pivotal finding rapidly established Polθ as a compelling therapeutic target in oncology. Therapeutic strategies exploiting Polθ synthetic lethality in tumors have been successfully reported, offering crucial theoretical support for its clinical translation (Figure 5).

Polθ is pivotal in guiding the cellular decision between HR and MMEJ for DSB repair.^103^ Polθ removes RPA from resected DNA ends, aids in microhomology annealing, and enhances MMEJ repair.^131^ Binding of a Polθ inhibitor to Polθ helicase domain prevents RPA displacement from resected DNA ends by inhibiting Polθ function. This leads to elevated BLM/EXO1-mediated end resection, steering the cell toward the HR pathway for repair. In HRD cells, excessive DNA end resection can lead to RPA accumulation and persistent DNA DSBs.^132^ ^,^ ^133^ Under these conditions, cells become highly dependent on Polθ to displace RPA, reducing RPA accumulation and utilizing MMEJ as a compensatory mechanism to repair DNA damage and maintain cell viability. Pharmacological inhibition of Polθ in this context results in sustained RPA accumulation, failure of compensatory repair, and ultimately cell death.^99^

In a mouse xenograft model of BRCA1-deficient breast cancer, novobiocin treatment significantly decreased tumor volume and nearly tripled overall survival compared to the control group.^99^ *Ex vivo* studies showed that novobiocin significantly decreased cell viability in BRCA1/2-deficient cells compared to wild-type controls.^99^ The clinical application of PARP inhibitors in cancer treatment has increasingly led to the emergence of tumor cell resistance.^94^ ^,^ ^134^ Based on the dual synthetic lethality strategy, Polθ inhibitors offer a novel potential strategy to overcome PARP inhibitor resistance.^117^ In tumors with HRD, MMEJ is crucial for DNA double-strand break repair. Inhibiting Polθ can boost the antitumor effectiveness of PARP inhibitors by triggering a synthetic lethal effect in cells deficient in homologous recombination.^116^ ^,^ ^117^ Research results demonstrated that in HRD cells, the olaparib and novobiocin combination was significantly more potent than olaparib alone.^99^ Novobiocin lowered the half-maximal inhibitory concentration (IC50) of PARP inhibitors in cells deficient in BRCA1 and FANCF. Research showed that Polθ protein expression was notably increased in HRD cells and was strongly associated with cellular sensitivity to novobiocin. These findings were further validated in patient-derived xenograft models with HR deficiency.

ART558's inhibition of Polθ was similar to the effects of Polθ expression reduction via siRNA. ART558 exhibited synthetic lethality in HRD cells. HRD cells exhibit markedly greater sensitivity to ART558 compared with HR-proficient cells. Following ART558 treatment, sustained γH2AX accumulation and persistent DNA damage were observed in HR repair-deficient cells, which subsequently induced apoptosis.^135^ Furthermore, high sensitivity to ART558 was observed in HRD breast cancer organoid models. When the repair function was restored in these deficient cells, their sensitivity to ART558 was significantly reduced, indicating the potential therapeutic application of ART558 in HRD tumors (Figure 5). Similar to novobiocin, ART558 could also overcome PARP inhibitor resistance in HRD cells through a dual synthetic lethality mechanism, providing a novel strategy to address the clinical challenge of PARP inhibitor resistance.^108^

### 6.3. Synthetic lethality between Polθ and NHEJ

MMEJ is a vital compensatory mechanism for NHEJ. Therefore, when the NHEJ function is impaired, Polθ expression within cells is elevated to compensate for DDR. Patterson-Fortin et al. found that inhibition of NHEJ through a DNA-dependent protein kinase catalytic subunit (DNA-PKcs) inhibitor significantly elevated Polθ expression.^83^ Genetic knockout of DNA-PKcs in cancer cells also significantly increased Polθ expression and made the cells highly sensitive to novobiocin, demonstrating a synthetic lethal interaction between the two repair pathways. Polθ and DNA-PKcs inhibition induced toxic levels of hyper-resection at DSB ends, accompanied by a significant increase in p‑RPA and γH2AX levels, signifying substantial accumulation of DNA damage.^83^ In cellular tumor antigen p53 (TP53) knockout cells, Polθ expression was markedly increased, and sensitivity to novobiocin was enhanced. When a DNA-PKcs inhibitor was administered under these conditions, Polθ expression was further upregulated, and cells became even more sensitive to novobiocin, indicating that combining a DNA-PKcs inhibitor and novobiocin can significantly improve therapeutic efficacy. This finding was validated in TP53-mutant ovarian cancer organoids, where the combined administration of novobiocin and DNA-PKcs inhibitor resulted in the accumulation of hyper-resected DSB ends, exerted toxicity on the organoids, and consequently reduced their viability.^83^ In mouse models, the combination of novobiocin and a DNA-PKcs inhibitor demonstrated significantly greater tumor-suppressive effects than novobiocin alone.^83^ Inhibiting both NHEJ and MMEJ resulted in excessive end resection and significant DNA damage accumulation, culminating in cell death (Figure 5).

Additionally, Patterson‑Fortin et al. confirmed that cells treated with the DNA-PKcs inhibitor remained sensitive to ART558, suggesting that ART558 could specifically target Polθ and exert a profound therapeutic effect.^83^ ART558, a specific small-molecule inhibitor of Polθ, demonstrated significant therapeutic potential in tumor cells lacking nonhomologous end joining repair. Furthermore, Rashmi J. Kumar et al. demonstrated that inhibiting DNA‑PKcs caused TP53‑deficient cancer cells to rely more heavily on Polθ‑mediated end‑joining repair for survival.^136^ Simultaneous inhibition of DNA-PKcs and Polθ enhanced radiosensitivity in cancer cells lacking TP53. Therefore, combined targeting of DNA‑PKcs and Polθ represented a promising strategy for treating TP53‑deficient tumors.

### 6.4. Synthetic lethality of Polθ with other DNA repair proteins

William Fried et al. demonstrated that dual inhibition of Polθ and PARP markedly increased cytotoxicity in HRD cancer cells (Figure 5). Furthermore, Xixi Lin et al. reported that combining a PARP inhibitor with Polθ inhibitors such as ART558 or novobiocin produced a notable radiosensitizing effect, indicating that dual inhibition of Polθ and PARP was a viable therapeutic strategy.^137^ Katherine Sullivan-Reed et al. found that targeting Polθ alongside PARP1 or RAD52 concurrently amplified the synthetic lethal effect in HRD leukemia cells.^117^ The concurrent application of a Polθ inhibitor with either a PARP or RAD52 inhibitor resulted in increased DNA DSB damage and enhanced cytotoxicity in HRD leukemia and myeloproliferative neoplasm cells. Similarly, Gabriela Barszczewska-Pietraszek et al. reported that dual Polθ inhibition with PARP1 or RAD52 significantly reduced glioblastoma cell viability and effectively induced apoptosis while having minimal impact on normal human astrocytes.^116^ The results suggested that inhibiting both Polθ and either PARP1 or RAD52 could effectively target and kill glioblastoma cells via a synthetic lethal mechanism (Figure 5). Weimin Fang et al. developed a nanosystem for DNA repair inhibitors, which enabled the codelivery of the PARP inhibitor olaparib and Polθ inhibitor novobiocin, ensuring effective accumulation and precise release of both drugs at the tumor site.^138^ Experiments demonstrated that the nanosystem effectively reduced breast cancer cell viability, increased DNA damage, and induced apoptosis, suggesting its potential as a therapeutic strategy for breast cancer.

Yi-Ru Pan et al. reported that in gemcitabine-resistant biliary tract cancer cells, Polθ deficiency sensitized cells to ataxia-telangiectasia mutated serine/threonine kinase (ATM) inhibitors.^139^ Combined Polθ deficiency and ATM inhibition resulted in substantial DNA damage, thereby enhancing tumor cell death. These findings indicated that targeting the synthetic lethal relationship between Polθ and ATM by applying ATM inhibitors in Polθ-deficient cells represented a potential therapeutic strategy for tumors (Figure 5). Grace Oh et al. confirmed in pancreatic cancer research that Polθ knockout exhibited a synthetic lethal effect with mutations in the DDR gene ATM.^140^ Additionally, Shibo Li et al. discovered that under high replication stress, the simultaneous inactivation of ATM and RAD3-related serine/threonine kinase (ATR) and Polθ synergistically eradicated tumor cells while maintaining low toxicity to normal cells (Figure 5). This finding provided new insights and strategies for cancer therapies.^141^ ^,^ ^142^

## 7. Discussion

Polθ is closely associated with tumorigenesis and progression. Compared with normal cells, the Polθ expression level is significantly elevated in tumor cells. Elevated Polθ expression leads to genomic instability and mutation accumulation, thereby promoting tumor development.^19^ ^,^ ^21^ Moreover, inhibition of Polθ effectively suppresses tumor cell growth and induces tumor cell death, an effect particularly significant in cells with defective DNA DSB repair function.^24^ ^,^ ^40^ Targeting Polθ has become a promising cancer therapeutic strategy with clinical translation potential, due to the discovery of synthetic lethal interactions between Polθ and DDR pathways,^125^ the development of various Polθ inhibitors,^102^ and the initiation of related clinical trials.

### 7.1. Core challenges restricting the clinical translation of Polθ-targeted therapy

There are some core challenges in the clinical translation of Polθ-targeted therapy. First, Polθ expression levels can indicate cellular sensitivity to Polθ inhibitors,^99^ yet this single marker cannot accurately stratify patient response in clinical practice, which forms the primary biomarker bottleneck of this targeted therapy. First, Polθ protein expression alone cannot fully reflect the functional dependency of tumor cells on MMEJ. Second, there is a lack of dynamic pharmacodynamic biomarkers to monitor real-time drug response during treatment. Third, no reliable biomarkers are established to predict the synergistic effect of combination regimens. These problems hinder the design of stratified clinical trials and the guidance of individualized medication.

With the advancement of multiple Polθ inhibitors entering Phase I/II clinical trials, preclinical models have revealed multiple intrinsic and acquired resistance mechanisms, which represent a major translational challenge. When MMEJ is suppressed, tumor cells may activate alternative repair routes to bypass drug pressure. Acquired drug-resistant cell lines may show persistent amplification of the POLQ gene locus or enhanced transcription mediated by oncogenic signaling pathways. In addition, missense mutations in the helicase ATP-binding pocket or polymerase catalytic pocket can directly reduce Polθ inhibitors' binding affinity, generating target-site resistance. These potential resistance mechanisms may reduce the antitumor effect of Polθ inhibitors and hinder the clinical translation of Polθ inhibitors.

Because of Polθ’s indispensable physiological roles, long-term Polθ inhibition causes potential off-tumor toxic risks.^44^ ^,^ ^48^ Germ cells and hematopoietic stem cells maintain low but detectable Polθ expression to resolve replication-associated DSBs. High-dose Polθ inhibitors may induce reproductive toxicity and hematopoietic suppression. MMEJ acts as a backup protective repair pathway when HR/NHEJ are temporarily suppressed in normal proliferative cells. Chronic Polθ blockade may lead to persistent unrepaired DSBs, increasing somatic mutation load and potential secondary tumor risk after long-term maintenance therapy. In addition, the dense tumor stroma reduces intratumoral drug enrichment, which requires high systemic dosage that exacerbates systemic side effects. These tissue safety issues must be taken into account during the clinical application of Polθ inhibitors.

In addition, there are gaps in research regarding many aspects of Polθ. First, the regulatory mechanisms of Polθ expression and its oncogenic molecular mechanisms are relatively limited. Second, existing research demonstrated that tumors that responded to immunotherapy were associated with Polθ expression,^140^ ^,^ ^143^ offering a new direction for the combined application of Polθ inhibitors and immunotherapy, though its efficacy requires further validation. Third, Polθ inhibitors developed thus far require additional clinical trials to demonstrate their safety and efficacy. Fourth, research on synthetic lethal interactions between Polθ and other DNA repair proteins is needed to enhance the understanding of Polθ and expand the potential applications of Polθ inhibitors.^122^ ^,^ ^123^ ^,^ ^128^ ^,^ ^144^

### 7.2. Conclusions and future research directions

Given that no Polθ inhibitor has been approved for clinical use to date, we further elaborate on multiple translational strategies to accelerate the clinical translation of Polθ-targeted therapeutics. First, structural optimization of small-molecule Polθ inhibitors could improve pharmacokinetic properties and target selectivity, reducing off-target genotoxicity toward normal tissues. Second, preclinical validation based on patient-derived xenografts and tumor organoids facilitates the identification of predictive biomarkers to screen HRD patient populations suitable for Polθ inhibition. Third, combinatorial regimens combining Polθ inhibitors with PARP inhibitors, chemotherapy, or immune checkpoint blockade represent a promising strategy to overcome the problem of drug resistance and enhance antitumor efficacy, which can guide the design of subsequent phase I/II clinical trials. Collectively, these multi-dimensional translational approaches may address major bottlenecks hindering the clinical advancement of Polθ inhibitors and lay a foundation for future targeted therapy development.

At present, all Polθ clinical candidates belong to traditional enzyme inhibitors, which rely on continuous occupancy of Polθ helicase or polymerase active pockets to block MMEJ repair function. However, this occupancy-driven pharmacological mechanism inevitably brings two universal resistance defects: missense mutations at the binding pocket reduce ligand affinity, and sustained POLQ transcriptional amplification increases intracellular Polθ protein abundance to offset inhibitory effects. Proteolysis targeting chimeras (PROTACs) technology, as an emerging targeted protein degradation modality, provides a transformative solution to overcome the above limitations.^145^

Unlike inhibitors that only block enzymatic activity, PROTACs exploit the intracellular E3 ubiquitin ligases to facilitate degradation of Polθ through the ubiquitin-proteasome system in a cyclic catalytic manner, thoroughly abolishing MMEJ repair capacity rather than partial functional suppression.^146^ Its unique catalytic mode brings two core advantages against drug resistance: PROTACs only require weak binding to any surface domain of Polθ to initiate degradation. Even if missense mutations occur at the small-molecule inhibitor binding site, PROTACs can still recruit E3 ligase through alternative binding regions to degrade mutant Polθ protein, completely avoiding target-site resistance caused by active site mutations; small-molecule inhibitors require high sustained intracellular concentrations to inhibit excessive Polθ protein in resistant clones with POLQ amplification. In contrast, PROTAC acts as a catalytic recyclable molecule. After degrading one Polθ protein, it dissociates and participates in the next round of ubiquitination degradation cycle at low effective concentration, efficiently clearing overexpressed Polθ protein.^147^ In addition, full-length Polθ depletion induced by PROTACs simultaneously eliminates both helicase and polymerase enzymatic activities, achieving more thorough blockade of MMEJ compared with single-domain small-molecule inhibitors. Polθ PROTACs research is currently in the early exploratory stage of preclinical development. Future studies are needed to accelerate the rational design and in vivo pharmacodynamic evaluation of Polθ PROTAC degraders, and to further explore the therapeutic potential of PROTAC degraders in combination with PARP inhibitors, chemotherapy, and immune checkpoint blockade.

In summary, precise screening of tumor patients benefiting from Polθ-targeted therapy, breakthroughs in overcoming drug resistance, and optimization of safety profiles will jointly promote the clinical translation of Polθ-targeted therapy, providing a novel targeted treatment option for DNA repair-deficient malignant tumors with limited existing therapeutic strategies.

## Data Availability

No datasets were generated or analyzed during the current study.

## Abbreviations

ALDH1A1: Aldehyde dehydrogenase 1A1
ATM: Ataxia-telangiectasia mutated serine/threonine kinase
ATR: ATM and RAD3-related serine/threonine kinase
BRCA1: Breast cancer susceptibility gene 1
BRCA2: Breast cancer susceptibility gene 2
CtIP: CtBP-interacting protein
DDR: DNA damage repair
DNA-PKcs: DNA-dependent protein kinase catalytic subunit
DSBs: Double-strand breaks
EWS-FLI1: Ewing sarcoma-FLI1
EWSR1: Ewing sarcoma R1
FANCD2: Fanconi anemia complementation group D2
FTO: Fat mass and obesity-associated protein
HCC: Hepatocellular carcinoma
HR: Homologous recombination
HRD: HR-deficient
IC50: Half-maximal inhibitory concentration
LIG1: DNA ligase I
LIG3: DNA ligase III
LIG4: DNA ligase IV
MLH1: MutL homolog 1
MMEJ: Microhomology-mediated end joining
MMR: Mismatch repair
MRN: MRE11-RAD50-NBS1
MSH2: MutS protein homolog 2
MSH3: MutS protein homolog 3
NF-κB: Nuclear factor kappa B
NHEJ: Nonhomologous end-joining
PARP1: Poly (ADP-ribose) polymerase 1
PLK1: Polo-like kinase 1
PROTACs: Proteolysis targeting chimeras
POLθ: DNA polymerase theta
RHINO: RAD1-interacting nuclear orphan protein 1
RPA: Replication protein A
SQSTM1: Sequestosome-1
SsDNA: Single-strand DNA
S6K: Ribosomal protein S6 kinase
TP53: Cellular tumor antigen p53
XRCC1: Protein X-ray repair cross-complementing 1
XRCC4: Protein X-ray repair cross-complementing 4
ZEB1: Zinc finger E-box binding homeobox 1
